# Genetic diversity and parasite facilitated establishment of the invasive signal crayfish (*Pacifastacus leniusculus*) in Great Britain

**DOI:** 10.1002/ece3.4235

**Published:** 2018-07-30

**Authors:** Chloe Victoria Robinson, Carlos Garcia de Leaniz, Joanna James, Joanne Cable, Pablo Orozco‐terWengel, Sofia Consuegra

**Affiliations:** ^1^ Swansea University Swansea Wales UK; ^2^ Cardiff University Cardiff Wales UK; ^3^ Environment Agency Brampton UK

**Keywords:** *Aphanomyces astaci*, crayfish plague, invasive species, microsatellite, population genetics, signal crayfish

## Abstract

Successful establishment of non‐native species is strongly influenced, among other factors, by the genetic variation of founding populations, which can be enhanced by multiple introductions through admixture. Coexisting pathogens can also facilitate the establishment of non‐native species by detrimentally impacting on the native fauna acting as novel weapons. The signal crayfish (*Pacifastacus leniusculus*) is a highly invasive species, which has caused mass declines of native crayfish in Europe through displacement and transmission of the oomycete *Aphanomyces astaci* (crayfish plague), which is typically lethal to native European crayfish. However, whether *Aphanomyces astaci* may have facilitated the invasion of the signal crayfish is not known. We estimated the genetic diversity at microsatellite DNA loci, effective population size, and potential origins of seven infected and noninfected signal crayfish populations in Europe and one founder population in North America. Approximate Bayesian computation analysis and population structuring suggested multiple host introductions from diverse source populations, as well as higher heterozygosity among infected than uninfected populations, which could reflect a fitness advantage. Low effective population size, moderate heterozygosity, and lack of isolation by distance suggest that some invasive signal crayfish populations may not be fully established or that their genetic diversity may have been reduced by eradication attempts.

## 1 INTRODUCTION

Non‐native species are often vehicles for the introduction of novel pathogens (Miaud et al., 2016; Randolph & Rogers, 2010) which, as important drivers of evolution, can impact the structure and composition of native communities (Altizer, Harvell, & Friedle, 2003) and facilitate invasion success (Andreou, Arkush, Guégan, & Gozlan, 2012; Vilcinskas, 2015). Pathogens carried by non‐native species can act as novel weapons on invaded communities (Price et al., 1986) and cause host‐switching (Lymbery, Morine, Kanani, Beatty, & Morgan, 2014; Peeler, Oidtmann, Midtlyng, Miossec, & Gozlan, 2011). The squirrelpox virus, for example, has facilitated the invasion of the North American gray squirrel (*Sciurus carolinensis*) in Europe (Collins et al., 2014), while the crayfish plague (caused by the oomycete *Aphanomyces astaci*) is expected to have played a similar role with the North American signal crayfish (*Pacifastacus leniusculus*; see Edgerton et al., 2004; Kaldre, Paaver, Hurt, & Grandjean, 2017). In both cases, these novel pathogens threaten the survival of phylogenetically close indigenous species, like the red squirrel (*Sciurus vulgaris*) and two native crayfishes, the white‐clawed crayfish, *Austropotamobius pallipes* and the noble crayfish, *Astacus astacus,* respectively. Yet, the extent to which novel pathogens drive invasion success is controversial (Blackburn & Ewen, 2017) and understanding why some species become established while others fail to do so remains a key question in invasion biology (Davis, 2009). Propagule pressure, phenotypic plasticity, and standing genetic diversity of founder populations are some of the most crucial factors that determine invasion success (Kolar & Lodge, 2001; Lee, 2002; Souty‐Grosset, Grandjean, & Renault, 2015; Vanhaecke et al., 2015). In general, newly introduced populations tend to lose genetic diversity due to founder effects (Dlugosch & Parker, 2008). Some of these bottlenecked populations successfully disperse and establish despite their low genetic diversity. For example, the spiny‐cheek crayfish (*Orconectes limosus*) is currently widespread across Europe despite having gone through a large bottleneck following a single introduction event of 90 individuals in 1890 (Filipová, Grandjean, Lieb, & Petrusek, 2011). In some cases, multiple introductions from different sources increase genetic diversity through admixture and facilitate establishment and dispersal (Allendorf & Lundquist, 2003; Consuegra, Phillips, Gajardo, & Garcia de Leaniz, 2011; Prentis, Wilson, Dormontt, Richardson, & Lowe, 2008).

Signal crayfish have been farmed across Great Britain since the 1970s (EA 2002) and accidental escapes, as well as deliberate stocking, have resulted in a number of populations becoming established in the wild (Alderman & Wickins, 1996; Griffiths, Collen, & Armstrong, 2004; Holdich 2003). Signal crayfish are much larger and more aggressive than their native counterparts, the white‐clawed crayfish, which they typically displace (Alderman & Wickins, 1996; Dunn et al., 2012; Griffiths et al., 2004; Peay, Guthrie, Spees, Nilsson, & Bradley, 2009). In addition to being displaced through competitive interactions, the white‐clawed crayfish is highly susceptible to crayfish plague infection, which is asymptomatically carried predominately by the signal crayfish (Bubb, Thom, and Lucas 2006; Dunn et al., 2012; Grandjean et al., 2017; Maguire et al., 2016) and is often 100% lethal to infected native crayfish (Edgerton et al. 2004).

Signal crayfish appears to have dispersed rapidly across Great Britain over the last 30 years (James, Slater, & Cable, 2014; James et al., 2017) and carry a particularly virulent strain of *A. astaci*, which has caused mass mortalities of *A. pallipes* in several European populations (Collas et al., 2016; Grandjean et al., 2014, 2017). However, the extent to which its current distribution has been facilitated by multiple introductions (Filipová, Holdich, Lesobre, Grandjean, & Petrusek, 2009) and/or by the presence of *A. astaci* is unclear. In addition, some populations have been subjected to control measures, mainly through the mechanical removal of thousands of crayfish, but the impact of these control measures is difficult to assess (Freeman, Turnbull, Yeomans, & Bean, 2010). Here, we compared the genetic diversity, effective population size, and potential origin of seven signal crayfish populations with different plague infection status and assessed the relative roles of the crayfish plague and multiple introductions in the establishment and dispersal of invasive crayfish in Great Britain.

## 2 METHODS

### 2.1 Study sites and sample collection

**Figure 1:**
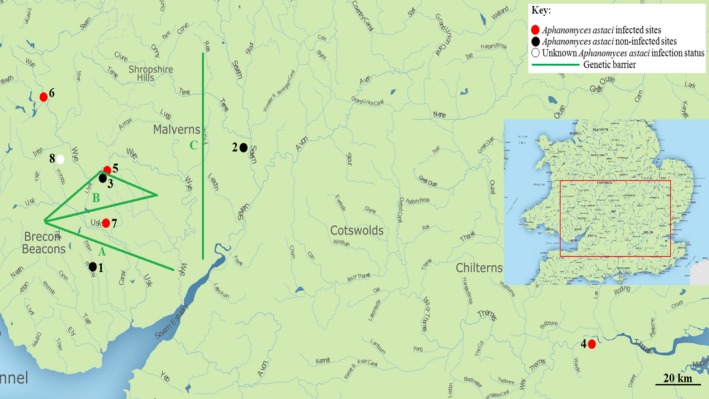
Map of UK sampling sites for *Pacifastacus leniusculus*, infection status, and the three highly significant breaks in genetic continuity generated by BARRIER in relation to sample sites (1 = Sirhowy, 2 = Lugg, 3 = Dderw, 4 = Lea, 5 = Bachowey, 6 = Mochdre, 7 = Gavenny, 8 = Pant‐y‐Llyn)

American signal crayfish were collected using baited crayfish traps (checked every 24 hrs) and hand netting (James et al., 2017), from five sites in Wales (Sirhowy, Dderw, Bachowey, Mochdre, and Gavenny) and two sites in England (Lugg and Lea) between May and September 2014 and one site (Pant‐y‐Llyn) in 2016 (Figure 1; Table 1). In addition, 30 crayfish were collected from a native population with unknown infection status in Oregon (US) as a reference for genetic diversity. The crayfish plague pathogen had not been detected at sites 1 (Sirhowy), 2 (Lugg) and 3 (Dderw), but had been isolated from crayfish at the remaining sites (James et al., 2017). Crayfish were collected under NRW Permits NT/CW065‐C‐652/5706/01 and NT/CW081‐B‐797/3888/02.

**Table 1:** Sample site information for all nine populations of *Pacifastacus leniusculus* sampled in Great Britain, including site name, latitude and longitude, site type and origin, catchment, crayfish plague infection status, and number of crayfish collected per site

Site No.	Site name (Country)	Latitude	Longitude	Site type (Origin)	Catchment	Infection status	No. of crayfish collected
1	Sirhowy (GB)	51.61628	−3.138935	Stream (Natural)	Usk	Noninfected	30
2	Lugg (GB)	52.10007	−2.420102	River (Natural)	Wye	Noninfected	30
3	Dderw (GB)	52.01450	−3.152826	Pond (Manmade)	Wye	Noninfected	30
4	Lea (GB)	51.47595	−0.043186	River (Natural)	Thames	Infected	37
5	Bachowey (GB)	52.06341	−3.135126	River (Natural)	Wye	Infected	19
6	Mochdre (GB)	52.30137	−3.520527	Stream (Natural)	Wye	Infected	19
7	Gavenny (GB)	51.82175	−3.012968	River (Natural)	Usk	Infected	30
8	Pant‐y‐Llyn (GB)	52.10932	−3.405192	Pond (Manmade)	Wye	Unknown	32
9	Oregon (USA)	44.55362	−123.2539	Stream (Natural)	Willamette	Unknown	19

### 2.2 DNA extraction and amplification

Total genomic DNA was extracted from each crayfish from a section of tail fan, soft abdominal cuticle and walking leg tissue using the DNeasy Tissue Kit (Qiagen, Sussex, UK) following the manufacturer's instructions (James et al., 2017). A total of 214 crayfish were analyzed using nine microsatellites (Table 2), in three separate multiplex reactions (Azuma, Usio, Korenaga, Koizum, & Takamura, 2011; Froufe et al., 2015). Extracted DNA was analyzed for quantity and quality using a Nanodrop 2000 (Thermo Fisher Scientific Inc., USA) and approximately 8 μg were used for amplification using the Qiagen Multiplex PCR kit, following the Qiagen multiplex reaction protocol (Qiagen) in a total volume of 12 μl. Each reaction consisted of the concentrations of primers detailed in (Froufe et al., 2015; Supporting InformationTable S1), with the exception of Scop31 (forward and reverse), which was reoptimized at 1 μM.

**Table 2:** Summary statistics for each population of *Pacifastacus leniusculus*. *N* = number of individuals, *N*_A_ = mean number of alleles, *N*_EF_ = mean number of effective alleles, *N*_e_ = effective population size, *N*_PA_= mean number of private alleles, *H*_O_ = mean observed heterozygosity, *H*_E_ = mean expected heterozygosity, *H*_L_ = mean homozygosity by locus, *F*_IS_ = mean fixation index

Population		*N*	*N*_A_	*N*_EF_	*N*_e_	*N*_PA_	*H*_O_	*H*_E_	*H*_L_	*F*_IS_
Sirhowy	Mean	30	4.222	2.460	12.300	0.000	0.397	0.569	0.607	0.309
	*SE* ±		0.795	0.195	8.000	0.000	0.076	0.041	0.030	0.121
Lugg	Mean	30	5.667	3.703	26.400	0.111	0.364	0.541	0.564	0.381
	*SE* ±		1.280	0.890	17.000	0.111	0.112	0.117	0.032	0.105
Dderw	Mean	30	4.111	2.248	19.900	0.111	0.273	0.464	0.676	0.349
	*SE* ±		0.790	0.301	11.200	0.111	0.079	0.090	0.030	0.116
Lea	Mean	37	7.778	4.552	76.500	0.444	0.426	0.628	0.516	0.328
	*SE* ±		1.392	1.054	47.000	0.242	0.099	0.092	0.022	0.103
Bachowey	Mean	19	5.000	3.090	29.900	0.222	0.398	0.577	0.552	0.307
	*SE* ±		0.772	0.527	14.600	0.147	0.080	0.079	0.036	0.087
Mochdre	Mean	19	6.778	4.108	42.800	0.111	0.427	0.653	0.525	0.367
	*SE* ±		1.222	0.773	22.100	0.111	0.104	0.072	0.040	0.117
Gavenny	Mean	30	8.556	5.445	90.400	0.389	0.441	0.687	0.519	0.350
	*SE* ±		1.730	1.430	49.500	0.564	0.073	0.073	0.035	0.081
Pant‐y‐Llyn	Mean	32	5.444	3.449	14.200	0.556	0.350	0.574	0.594	0.428
	*SE* ±		1.029	0.729	10.000	0.338	0.089	0.089	0.037	0.104
Oregon	Mean	19	7.222	4.191	32.600	1.556	0.374	0.588	0.502	0.404
	*SE* ±		1.623	8.972	18.700	0.580	0.118	0.103	0.050	0.137

Amplification conditions consisted of a single‐cycle initial activation step of 15 min at 95°C followed by a touchdown PCR of eight cycles with a 30 s denaturation step at 94°C, a 90 s annealing step starting at 64°C and descending in 2‐cycle steps of 2°C (64, 62, 60, 58 and 56°C) and 90 s of extension at 72°C. Twenty‐four additional cycles of PCR were then run as above at an annealing temperature of 56°C followed by a single final extension cycle of 30 min at 60°C. Microsatellites were resolved on an Applied Biosystems ABI3130xl Genetic Analyser (Applied Biosystems, Sussex, UK), and fragment length was determined using the GeneScan 500–LIZ size standard and scored using GeneMapper v45.0 (Applied Biosystems).

### 2.3 Genetic analyses

MICRO‐CHECKER v2.2.3 (Van Oosterhout, William, Hutchinson, Wills, & Shipley, 2004) was used to assess presence of null alleles, large allele drop‐outs and scoring errors due to stuttering. GENALEX v6.5 (Peakall & Smouse, 2006) was used to estimate effective number of alleles (*N*_EF_) and the populations’ expected (*H*_E_) and observed heterozygosities (*H*_O_) respectively. Deviations from Hardy–Weinberg equilibrium and tests for linkage disequilibrium were investigated using GENEPOP online v4.0.10 (Rousset, 2008). Pairwise *F*_ST_ values and heterozygosity per locus were calculated using FSTAT v1.2 (Goudet, 1995). Analysis of Molecular Variance (AMOVA) among populations, among individuals and within individuals was calculated in ARLEQUIN v3.1 (Excoffier, Laval, & Schneider, 2005). Homozygosity by locus (*H*_L_), which weighs the contribution of each locus to the homozygosity index depending on their allelic variability, was estimated for each individual in Cernicalin v1.0 (Aparicio, Ortego, & Cordero, 2006). Effective population size was estimated using N_e_Estimator v2.01 (Do et al., 2014) for samples with a minimum of 19 individuals using the Linkage Disequilibrium method with allele frequencies larger than 0.02. For *H*_L_ analyses, both the Oregon and Pant‐y‐Llyn populations were excluded, as crayfish plague infection status and therefore PCR forming units (PFU) values for these individuals were unknown.

STRUCTURE v2.3.4 (Pritchard, Stephens, & Donnelly, 2000), was used to estimate the most likely number of genetic clusters in the data. The analysis was run under the admixture ancestry model, computing the proportion of the genome of each individual originating from each cluster (*K*). The number of clusters tested ranged from *K* = 1 to 9, with 20 repetitions for each *K* value, and 60,000 MCMC steps discarding the first 10,000 as burn‐in (Pritchard et al., 2000). The best fitting *K* value was estimated using StructureSelector (Li & Liu, 2017), which utilizes four alternative statistics (MEDMEDK, MEDMEAK, MAXMEDK, and MAXMEAK) to produce more accurate results for populations with uneven sample size.

BARRIER v2.2 (Manni, Guérard, & Heyer, 2004) was used to detect discontinuities in allelic frequencies between British crayfish populations based on genetic distance and geographical distance values using the Monmonier's maximum difference algorithm (Monmonier, 1973). Initially one data matrix containing pairwise *F*_ST_ values was imported in BARRIER to detect genetic barriers across all populations. Eight data matrices were then imported into BARRIER containing pairwise *F*_ST_ values per locus to assess the number of loci supporting each barrier and test for barrier robustness’ (Manni et al., 2004).

The most likely scenario of colonization for UK populations was estimated using and Approximate Bayesian Computation approach implemented in the software DIYABC v2.1.0 (Cornuet et al., 2014). For this analysis the Lea, Mochdre, and Gavenny populations were grouped into one genetic group (pool 1) based on *F*_ST_ values and similarity of genetic clusters from the STRUCTURE analysis and the remaining populations were analyzed as separate populations. Three scenarios of colonization were tested (Figure 3): Scenario 1 – simultaneous divergence (null hypothesis), Scenario 2 – simultaneous divergence of Sirhowy, pool 1, Lugg, Bachowey and Dderw followed by divergence of Pant‐y‐Llyn from Bachowey, Scenario 3 – simultaneous divergence of Sirhowy, pool 1, Lugg, Bachowey and Dderw followed by admixture of pool 1 with Bachowey to produce the Pant‐y‐Llyn population. Default settings were used for mutation rates (generalized stepwise mutation model (Estoup, Jarne, & Cornuet, 2002) with a uniform prior distribution of mean mutation rate between 10^−4^ and 10^−3^, priors were set uniformly distributed, prior distribution of individual locus mutation rates were set between 10^−5^ and 10^−2^ following a Gamma distribution with mean determined by the mean mutation rate across loci. Effective population sizes were set between 10 and 2,500 for all populations. A total of 1,000,000 simulations per scenario (1,2,3) were generated from the parameters prior distributions. Mean gene diversity across loci and mean M index diversity across loci (one sample summary statistics) were calculated for each population. Pre‐evaluation of each scenario was carried out by generating Principal Component Analysis (PCA) plots based on summary statistics using 30,000 (1%) simulated data sets and the posterior distribution of the parameters was estimated using the logit function (Cornuet et al., 2014). For model checking, we performed a PCA using new simulated datasets (1,000,000 per scenario) drawn from the posterior distribution of parameters, which are also represented on the PCA. Two sample summary statistics were used in model checking (mean number of alleles, mean genic diversity, mean size variance, *F*_ST_, classification index, shared allele distance and (dμ)^2^ distance) to assess whether the observed data was included within the distribution of the predictive posterior parameters of the simulated data. Confidence in each scenario was obtained from the highest posterior probability using logistic regression, estimated by comparing the summary statistics from simulated and observed results, and from calculating type I and type II errors using 1000 simulated datasets (Cornuet et al., 2014).

Population heterozygosity and effective population size were compared between infected and noninfected populations using a Welch *t* test for unequal variances. We also modeled infection status (yes/no) and plague intensity (measured as density of plaque‐forming units, PFU) in individual crayfish using population of origin as a random factor and individual homozygosity (*H*_L_) as a predictor with either a binomial logit link (infection status) or a Gaussian link (plague intensity, measured as log(PFU+0.5) with the *lme4* package in R, version 3.3.2.

## 3 RESULTS

### 3.1 Host genetic diversity and population structuring

MICRO‐CHECKER results indicated that four microsatellites had significant evidence of null alleles (*p *=* *0.001), however results of repeated analyses (*F*_ST_, STRUCTURE) removing the affected microsatellites showed no obvious deviations from the results including all nine microsatellites (Supporting Information Table S2; Figure S1), therefore we carried out all subsequent analyses with all of them (Van Oosterhout et al., 2004). The nine microsatellite loci displayed moderate to high levels of polymorphism (*H*_E_ between 0.5 and 0.7) across all the sites. All populations displayed a degree of deviation from Hardy–Weinberg equilibrium (HWE) across various loci due to lower than expected *H*_E_. Of 81 Chi‐square tests conducted (one per locus) 37 showed a significant deviation from HWE (Supporting Information Table S3) after sequential Bonferroni correction. The across loci population tests of HWE showed that all populations deviated significantly from HWE, displaying a deficiency of heterozygosity (*p *<* *0.0001). Tests for linkage disequilibrium (LD) for each locus pair across all populations (Fisher's method) revealed only three significant associations of 36 pairwise comparisons after Bonferroni corrections, these were between LPL26 and LPL40, LPL6 and LPL45 and LPL26 and Scop9.

Across all populations, the mean number of alleles ranged from 4.11 to 8.56, with the Gavenny site having the highest mean number of alleles and the Sirhowy site the lowest across all populations sampled (Table 2). The mean expected heterozygosity (*H*_E_) across all populations ranged from 0.46 to 0.69 respectively. The mean effective number of alleles ranged from 2.25 in the Dderw site to 5.45 in the Gavenny site and for Lea the mean was 4.55. Across all loci, there was no significant difference in number of effective alleles (*N*_EF_) between populations (One‐way ANOVA, *F*_8,72_ = 1.496, *p* = 0.1739). Effective population size (*N*_e;_) ranged between 12.9 (Sirhowy) and 90.4 (Gavenny; Supporting Information Table S2) and, probably due to small sample size, confidence intervals were relatively large (3.9–28.6).

**Figure 2:**
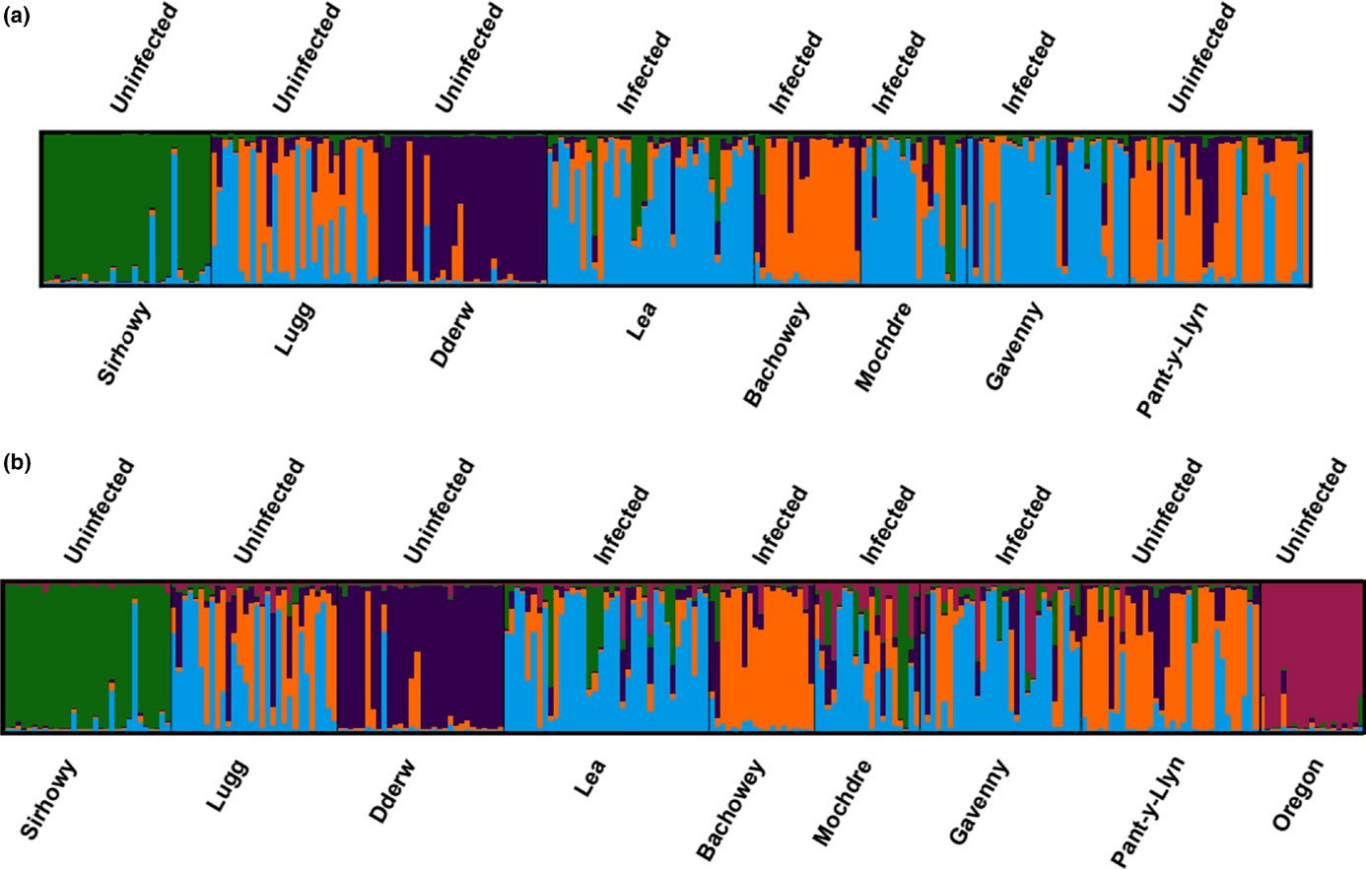
(a) STRUCTURE analysis for UK *Pacifastacus leniusculus* populations at *K* = 4 clusters; each bar represents an individual crayfish, the different colors represent different clusters and therefore indicate the proportion of each crayfish attributed to each cluster. Infection status is stated above the output and corresponding population names stated below for each population. (b) STRUCTURE analysis for all nine *P. leniusculus* populations at *K* = 5 clusters

The STRUCTURE and StructureSelector analyses indicated that *K* = 4 (Supporting Information Figure S2; Table S6) is the most likely number of clusters in the dataset for British populations only and *K* = 5 (Supporting Information Figure S3; Table S7) for the British populations plus Oregon (Figure 2a and b).

Pairwise population *F*_ST_ values were highly significant (after Bonferroni correction, *p *<* *0.00138), with the exception of the Mochdre and Gavenny sites, and the Mochdre and Lea sites (Table 3). Among the UK populations, the Dderw displayed the highest divergence, with the highest pairwise *F*_ST_ observed between the Dderw and Sirhowy populations (*F*_ST_ = 0.294, *p *<* *0.001). The AMOVA results for all sites indicated that variation among populations accounted for 15% of the genetic differentiation, while variance among individuals within populations accounted for 31% and the remaining 54% was due to intraindividual variation (Table 4). Most populations displayed a relatively high degree of admixture (average *Q* value 69%), apart from Sirhowy and Dderw (average *Q* value of 91%). Geographically distant populations (e.g., Lea and Mochdre) displayed high levels of genetic similarity despite distance, which was reflected in lack of IBD in the Mantel test for UK populations (*y* = 0.0009*x* + 17.053, *R* ^2^ = 0.0003, *p *>* *0.05).

**Table 3:** Pairwise *F*_ST_ values (below diagonal) and significance (above diagonal) for nine populations of invasive *Pacifastacus leniusculus* sampled in Great Britain

	Sirhowy	Lugg	Dderw	Lea	Bachowey	Mochdre	Gavenny	Pant‐y‐Llyn	Oregon
Sirhowy	0.000	a	a	a	a	a	a	a	a
Lugg	0.206	0.000	a	a	a	a	a	a	a
Dderw	0.294	0.138	0.000	a	a	a	a	a	a
Lea	0.162	0.041	0.139	0.000	a	NS	a	a	a
Bachowey	0.195	0.085	0.145	0.056	0.000	a	a	a	a
Mochdre	0.150	0.053	0.148	0.026	0.050	0.000	NS	a	a
Gavenny	0.155	0.056	0.129	0.029	0.063	0.017	0.000	a	a
Pant‐y‐Llyn	0.199	0.064	0.100	0.037	0.017	0.052	0.052	0.000	a
Oregon	0.334	0.337	0.410	0.273	0.312	0.253	0.258	0.315	0.000

Notes

Significance values for each pairwise comparison adjusted by sequential Bonferroni's corrections.

a *p *<* *0.00138.

**Table 4:** Results of analysis of molecular variance (AMOVA) for all nine populations of invasive *Pacifastacus leniusculus*, presenting the different sources of variation (among populations, among individuals, within individuals), degrees of freedom (*df*), sum of squared differences (SSD), variance components, percentage variation, and *p* value for each source

Source of variance	*df*	SSD	Variance components	Percentage variation	*p* Value
Among populations	8	235.163	0.475	14.892	<0.001
Among individuals	236	876.566	1.001	31.400	<0.001
Within individuals	245	419.500	1.712	53.708	<0.001

**Figure 3:**
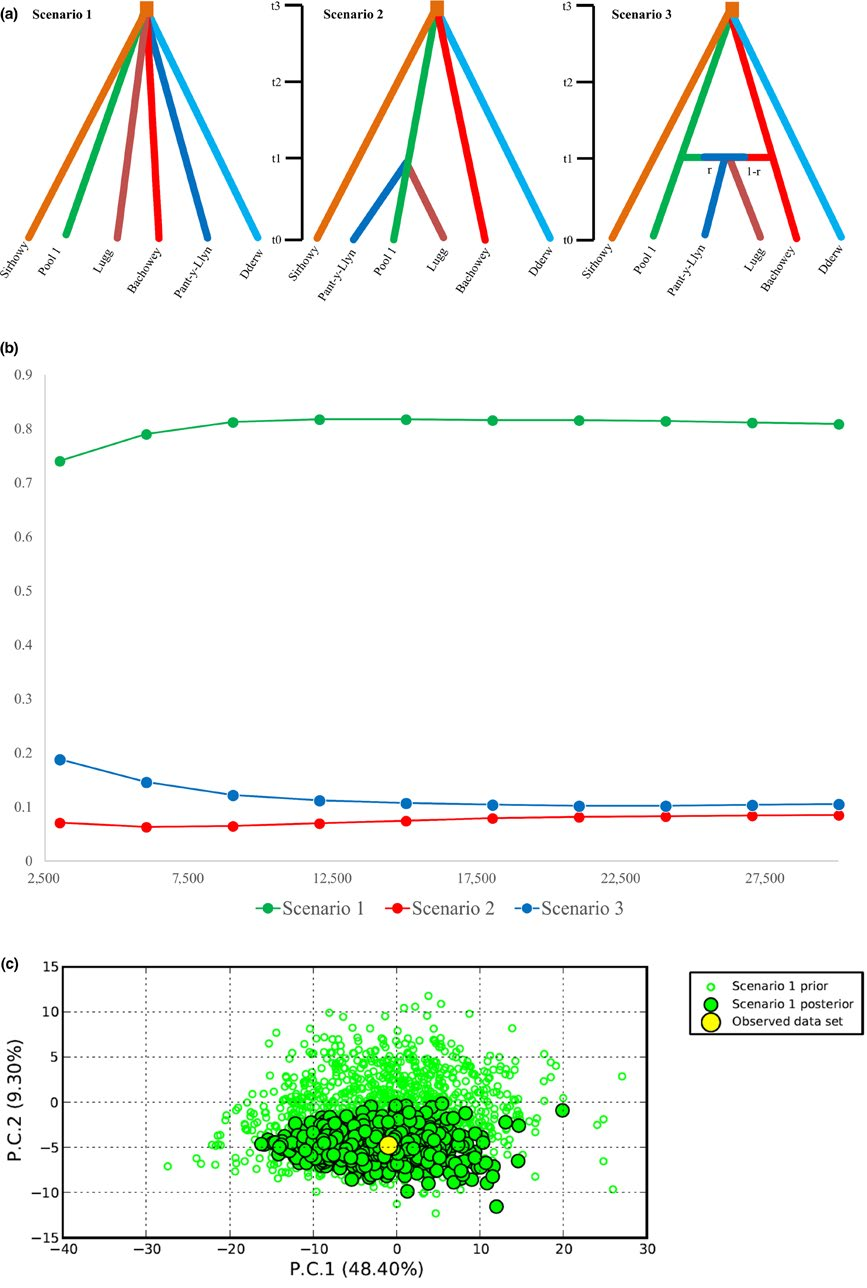
Three scenarios of signal crayfish colonization tested using approximate Bayesian computation analysis (ABC), (a) Simultaneous divergence (Scenario 1); simultaneous divergence of Sirhowy, Pool 1, Lugg, Bachowey, and Dderw, followed by divergence of Pant‐y‐Llyn from Bachowey (Scenario 2); and simultaneous divergence of Sirhowy, Pool 1, Lugg, Bachowey, and Dderw, followed by admixture of pool 1 with Bachowey to produce Pant‐y‐Llyn (Scenario 3), (b) Reliability of scenarios displayed through posterior probabilities as the logistic regressions, (c) Principal component analysis (PCA) showing the fit of posterior distributions for Scenario 1

Analysis of the most likely scenario of colonization suggested the simultaneous divergence of populations (Scenario 1), based on logistic regression and PCA results (Figure 3; Supporting Information Figure S5). Observed summary statistics did not deviate significantly from simulated statistics and scaled posteriors aligned well with priors (Supporting Information Table S4). According to this scenario, six main colonization events could have taken place; (1) Sirhowy; (2) Dderw; (3) Bachowey; (4) pool 1 (Lea, Mochdre, and Gavenny), (5) Lugg, (6) Pant‐y‐Llyn.

Populations with the lowest degree of admixture (Sirhowy, Bachowey and Dderw) did not have a significantly lower heterozygosity (*H*_E_ = 0.527) compared to populations which were more admixed (Lugg, Lea, Mochdre, Gavenny, Pant‐y‐Llyn; average *H*_E_ = 0.617; *df* = 4.4; Welch *t* test; *p* = 0.098). Results from BARRIER suggested that the most likely number of discontinuities in genetic connectivity was due to three barriers (Supporting Information Figure S4), the strongest division occurring between Sirhowy and all the other sites (Barrier a; Figure 1). The next largest discontinuity was observed between Dderw and surrounding populations (Barrier b; Figure 1), while the third barrier separated Lugg from the Welsh populations (Barrier c; Figure 1; site 2). All barriers were supported by seven of nine loci.

### 3.2 Aphanomyces astaci infection levels and population genetic diversity

Infected crayfish populations had a significantly higher mean expected heterozygosity than uninfected ones (*H*_E_ infected = 0.64, *SE* = 0.02; *H*_E_ uninfected = 0.52, *SE* = 0.02; Welch two sample *t* test = 3.509, *df* = 4.5, *p* = 0.019), while their effective population size did not differ significantly between them (average *N*_e_ infected = 59.90 *SE* = 24.48; average *N*_e_ uninfected = 19.73 *SE* = 5.51; Welch two sample *t* test = 2.36, *df* = 3.4, *p* = 0.06).

**Figure 4:**
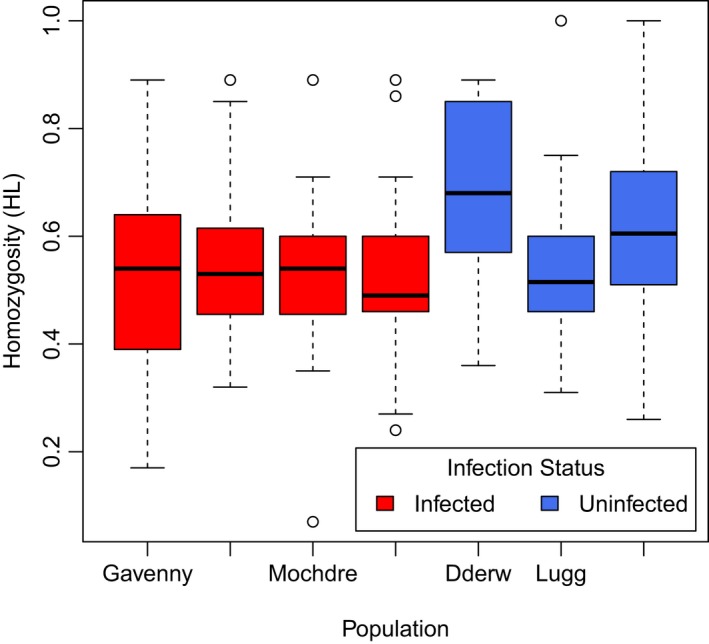
Homozygosity by locus (*H*_L_) values for each of the crayfish populations with known infection status

Mean *H*_L_ for each population ranged from 0.50 to 0.68 (0 being heterozygous and 1 being completely homozygous). Crayfish populations differed significantly in individual homozygosity (Figure 4; *F*_6,187_ = 3.71, *p* = 0.002), with infected populations having a significantly lower homozygosity by locus. Crayfish populations also differed significantly in plague infection loads (*F*_6,187_ = 38.27, *p *<* *0.001), but homozygosity did not explain the probability that an individual would be infected (*z* = 1.337, *p* = 0.181) or the intensity of infection (*t*_186.94_ = 0.874, *p* = 0.383) when controlling for population of origin (Supporting Information Table S5).

## 4 DISCUSSION

Signal crayfish represents an ideal species to test the roles of genetic diversity and pathogens as novel weapons on invasion success, as the species is highly invasive throughout most of Europe, and Great Britain in particular. Its success has been attributed to preadaptation, aggressive behavior, niche plasticity, and the presence of the highly infectious *A. astaci* (Becking et al., 2015; Holdich, James, Jackson, & Peay, 2014; Hudina, Galić, Roessink, & Hock, 2011; James et al., 2014). Admixture between lineages could have also facilitated the establishment of this species, allowing populations to overcome founder effects and loss of genetic diversity (Kolbe et al., 2004; Rius & Darling, 2014), particularly when combined with high propagule pressure (Consuegra et al., 2011), but this had not been considered before. In Britain, the species has continued to spread despite management and control measures (Holdich et al., 2014).

The invasive signal crayfish populations we studied had small effective population sizes and low to moderate genetic diversity, despite having been established for more than 25 years (*c*. 25 generations), which is similar to what is observed in other invasive crayfish species populations, (e.g., the red swamp crayfish (*Procambarus clarkii*) in China (Yue, Li, Bai, Wang, & Feng, 2010), Mexico and Costa Rica (Belfiore & May, 2000; NBN 2009; Torres & Álvarez, 2012). It is possible that the low levels of heterozygosity observed are the result of recurrent translocations of small numbers of signal crayfish which may have resulted in founder effects (Gouin, Grandjean, & Souty‐Grosset, 2006). None of the populations were in Hardy–Weinberg equilibrium and all had lower than expected heterozygosity, which is consistent with founder effects. In addition, lack of isolation by distance and strong population structuring could be the result of multiple introductions from different sources (Le Roux & Wieczorek, 2008; Roman & Darling, 2007) or of small founder sizes followed by genetic drift and isolation. Eradication efforts over the last 10 years, such as the removal of *c*. 56,000 crayfish in the Bachowey (Abdelkrim, Pascal, & Samadi, 2007; WUF, 2012), could have also contributed to decreasing genetic diversity and increasing population structuring, but the documentation of these events is too scarce to draw any conclusions.

Evidence of four main genetic clusters in the introduced signal crayfish populations, the strong differentiation (*F*_ST_) values and the most likely colonization scenario support the assumption that current crayfish populations in Britain are not genetically homogenous, a phenomenon common in invasive species originating from different origins (Zalewski, Michalska‐Parda, Bartoszewicz, Kozakiewicz, & Brzeziński, 2010). The most likely colonization scenario for signal crayfish in the sites sampled in Britain suggested that populations most likely originated from six source populations with varying levels of genetic diversity, although some caution is warranted in the interpretation of the results due to the deviations from Hardy–Weinberg equilibrium. The observed spatial pattern of infected crayfish populations is best explained by considering numerous founder events and further colonization helped by human‐mediated dispersal. This corresponds well to the diversity of mitochondrial DNA previously observed for this species in Europe (including six different haplotypes in the British Isles), which lacks a geographical pattern and has been attributed to different introductions and secondary human‐mediated translocations (Petrusek, Filipova, Kozubíková‐Balcarová, & Grandjean, 2017), and would explain why some infected populations (Lea, Mochdre, and Gavenny) have a common genetic background, similar to what has been observed in the Czech Republic (Kozubíková et al., 2008). Infected crayfish populations had higher heterozygosity than uninfected populations which, if representative of whole genome heterozygosity, could represent higher fitness (Forstmeier, Schielzeth, Mueller, Ellegren, & Kempenaers, 2012; Reed & Frankham, 2003). The presence of infected and uninfected signal crayfish in close proximity (i.e., Dderw and Bachowey) could be a consequence of physical barriers and is important in relation to the conservation of endangered native crayfish populations, as invasive signal crayfish and native European crayfish can coexist in the absence of plague (Bubb, Thom, & Lucas, 2005; Diéguez‐Uribeondo, 2006; Filipová, Petrusek, Matasová, Delaunay, & Grandjean, 2013). Native crayfish tend to inhabit refugia in the headwaters of numerous catchments within Britain, some of which have tested positive for *A. astaci* downstream (Bubb et al., 2005; Filipová et al., 2013).

In summary, it is likely that human‐mediated dispersal has contributed to the numerous colonization events from a minimum of four genetic origins and further facilitated population expansion and succession of signal crayfish. Populations with *A. astaci* displayed higher heterozygosity, which could potentially be an indication of fitness benefits or a consequence of the absence of competition with native crayfish; however, physical and/or environmental barriers to dispersal may have additionally contributed to differences in *A. astaci* infection levels between populations as opposed to the varying genetic diversity of individual crayfish. Despite this species’ invasion success, low effective population size and levels of genetic diversity observed suggest that populations are either still establishing in Great Britain and have not yet overcome the effects of founder effects or have suffered a decrease in local genetic diversity as a result of invasive crayfish removal programs. The success of local management programs is difficult to assess, as crayfish populations are very difficult to eradicate by mechanical means (Freeman et al., 2010), and negative density dependence can improve the body condition of survivors (Moorhouse & Macdonald, 2011). However, our study suggests that genetic monitoring before and after physical removal of crayfish can provide measures of genetic diversity and effective population size that could be used to assess the population consequences of removal actions.

## CONFLICT OF INTEREST

None declared.

## AUTHOR CONTRIBUTIONS

SC & CVR designed the study; CVR performed the genetic analyses with advice from SC and POTW; JC & JJ contributed samples and information; CGL performed statistical analyses; and CVR, SC, and CGL wrote the manuscript, which was revised by of all the authors.

## DATA ACCESSIBILITY

Raw data (microsatellite genotypes per individual, parasite loads) will be archived in Dryad upon acceptance.
