# Supplementary material for: Genetic diversity and parasite facilitated establishment of the invasive signal crayfish (Pacifastacus leniusculus) in Great Britain
Source: Ecol Evol. 2018 Jul 30;8(18):9181–91. doi: 10.1002/ece3.4235 (PMC6194297; doi:10.1002/ece3.4235)

**Supplementary Information**

**Table S1.** Information on the microsatellites used in analysis (Froufe et al. 2015), including multiplexes used, primer sequences, fluorescent dyes, concentration of primer in PCR reaction (µm) and allele size range covered by each microsatellite (bp).

| Locus | Primer Sequence (5’–3’) | Fluorescent dye | Primer concentration (µm) | Allele size range (bp) |
| --- | --- | --- | --- | --- |
| Multiplex 1 |  |  |  |  |
| LPL6 | F: TGTCGGATCATAGTCGTCGT | VIC | 0.45 | 103-172 |
|  | R: GGCTCTCAGTAACCTACCAGG |  |  |  |
| LPL26 | F: AAATAAGACCCGACAAAGCG | FAM | 0.5 | 301-337 |
|  | R: ATGAGGAGCCGCAAGTGTAA |  |  |  |
| LPL40 | F: CAGTCGATATTTCTTTATATCCCTTCA | PET | 0.6 | 101-137 |
|  | R: CTGGTTCCAGATAGCAGCGT |  |  |  |
| Multiplex 2 |  |  |  |  |
| LPL15 | F: TGTCGGATCATAGTCGTCGT | PET | 0.7 | 95-173 |
|  | R: AGGCTCTCAGTAACCTACCAGG |  |  |  |
| LPL32 | F: AAAGCGGACAACATGGAAGT | FAM | 2.5 | 266-318 |
|  | R: GCCGCAAGTGTAAGCTGAA |  |  |  |
| LPL45 | F: TCTAGAAACAGACTGGTCTCATGG | FAM | 1 | 69-114 |
|  | R: CACCTACGGGCTATTCATGC |  |  |  |
| Multiplex 3 |  |  |  |  |
| Scop1 | F: GCCCTCTGCTTACTTTCTCAC | NED | 0.4 | 131-183 |
|  | R: CTCATGGAGTACGAGTCCAGA |  |  |  |
| Scop9 | F: GCTGAAATGGAGGGATGA | FAM | 0.4 | 140-170 |
|  | R: TGTGCCTTTTCTAAGCTGT |  |  |  |
| Scop31 | F: GATCTGGACGTCGACGTCTT | VIC | 1 | 184-256 |
|  | R: CCCTGTACCATTCATGATTG |  |  |  |

**Table S2.** Pairwise *F*_ST_ values (below diagonal) and significance (above diagonal) for all populations of *Pacifastacus leniusculus* sampled with ‘null’ microsatellites (Scop1, Scop9, Scop31 and LPL45) removed.

|  | Sirhowy | Lugg | Dderw | Lea | Bachowey | Mochdre | Gavenny | Pant-y-Llyn | Oregon |
| --- | --- | --- | --- | --- | --- | --- | --- | --- | --- |
| Sirhowy | 0.000 | * | * | * | * | * | * | * | * |
| Lugg | 0.261 | 0.000 | * | * | * | * | * | * | * |
| Dderw | 0.350 | 0.126 | 0.000 | * | * | * | * | * | * |
| Lea | 0.207 | 0.041 | 0.137 | 0.000 | * | * | - | * | * |
| Bachowey | 0.222 | 0.064 | 0.160 | 0.064 | 0.000 | * | * | - | * |
| Mochdre | 0.184 | 0.057 | 0.199 | 0.032 | 0.035 | 0.000 | - | * | * |
| Gavenny | 0.207 | 0.050 | 0.168 | 0.015 | 0.054 | 0.010 | 0.000 | * | * |
| Pant-y-Llyn | 0.228 | 0.031 | 0.083 | 0.027 | 0.020 | 0.040 | 0.028 | 0.000 | * |
| Oregon | 0.392 | 0.441 | 0.545 | 0.362 | 0.371 | 0.330 | 0.334 | 0.398 | 0.000 |

* *P* < 0.00138

**Table S3.** Genetic diversity values of nine *Pacifastacus leniusculus* populations for nine microsatellite loci. *N*_A_ Number of alleles; *N*_E_ Number of effective alleles; *H*_O_ Observed heterozygosity; *H*_E_ Expected heterozygosity; *HW* *P*-values for deviation of Hardy-Weinberg equilibrium; *F_IS_* Fixation index (positive value indicates homozygosity excess); N Number of samples. Significant values of deviation of HW after Bonferroni correction (*P* <0.000617) are indicated in bold.

|  |  | Microsatellite | | | | | | | | |
| --- | --- | --- | --- | --- | --- | --- | --- | --- | --- | --- |
| Population |  | LPL15 | LPL26 | LPL32 | LPL40 | LPL45 | LPL6 | Scop1 | Scop31 | Scop9 |
| 1 (Sirhowy) | *N*_A_ | 5 | 3 | 3 | 3 | 2 | 3 | 10 | 5 | 4 |
|  | *N*_E_ | 2.961 | 2.582 | 2.052 | 2.187 | 1.399 | 2.799 | 3.422 | 2.532 | 2.209 |
|  | *H*_O_ | 0.567 | 0.700 | 0.433 | 0.633 | 0.138 | 0.533 | 0.233 | 0.267 | 0.067 |
|  | *H*_E_ | 0.662 | 0.613 | 0.513 | 0.543 | 0.285 | 0.643 | 0.708 | 0.605 | 0.547 |
|  | *HW* | **0.000** | 0.567 | 0.571 | 0.195 | 0.005 | 0.548 | **0.000** | **0.000** | **0.000** |
|  | *F*_IS_ | 0.144 | -0.142 | 0.155 | -0.167 | 0.517 | 0.170 | 0.670 | 0.559 | 0.878 |
| 2 (Lugg) | *N*_A_ | 12 | 1 | 1 | 4 | 4 | 10 | 7 | 8 | 4 |
|  | *N*_E_ | 8.145 | 1.000 | 1.000 | 3.035 | 1.418 | 7.627 | 4.749 | 3.298 | 3.056 |
|  | *H*_O_ | 0.867 | 0.000 | 0.000 | 0.600 | 0.067 | 0.833 | 0.333 | 0.310 | 0.267 |
|  | *H*_E_ | 0.877 | 0.000 | 0.000 | 0.671 | 0.295 | 0.869 | 0.789 | 0.697 | 0.673 |
|  | *HW* | 0.764 | N/A | N/A | **0.000** | **0.000** | 0.740 | **0.000** | **0.000** | **0.000** |
|  | *F*_IS_ | 0.012 | N/A | N/A | 0.105 | 0.774 | 0.041 | 0.578 | 0.555 | 0.604 |
| 3 (Dderw) | *N*_A_ | 8 | 1 | 2 | 2 | 5 | 5 | 7 | 4 | 3 |
|  | *N*_E_ | 3.673 | 1.000 | 1.034 | 1.684 | 2.539 | 2.050 | 2.934 | 2.998 | 2.323 |
|  | *H*_O_ | 0.767 | 0.000 | 0.033 | 0.367 | 0.133 | 0.433 | 0.148 | 0.241 | 0.333 |
|  | *H*_E_ | 0.728 | 0.000 | 0.033 | 0.406 | 0.606 | 0.512 | 0.659 | 0.666 | 0.569 |
|  | *HW* | 0.415 | N/A | 0.926 | 0.595 | **0.000** | 0.862 | **0.000** | **0.000** | **0.000** |
|  | *F*_IS_ | -0.053 | N/A | -0.017 | 0.097 | 0.780 | 0.154 | 0.775 | 0.638 | 0.415 |
| 4 (Lea) | *N*_A_ | 11 | 4 | 4 | 5 | 4 | 10 | 14 | 13 | 5 |
|  | *N*_E_ | 6.294 | 1.578 | 1.360 | 2.903 | 1.250 | 5.764 | 8.813 | 9.425 | 3.579 |
|  | *H*_O_ | 0.865 | 0.216 | 0.189 | 0.730 | 0.108 | 0.811 | 0.286 | 0.444 | 0.189 |
|  | *H*_E_ | 0.841 | 0.366 | 0.265 | 0.656 | 0.200 | 0.827 | 0.887 | 0.894 | 0.721 |
|  | *HW* | 0.997 | **0.000** | **0.000** | 0.482 | **0.000** | 0.992 | **0.000** | **0.000** | **0.000** |
|  | *F*_IS_ | -0.028 | 0.410 | 0.286 | -0.113 | 0.459 | 0.019 | 0.678 | 0.503 | 0.737 |
| 5 (Bachowey) | *N*_A_ | 8 | 3 | 3 | 4 | 3 | 7 | 7 | 8 | 3 |
|  | *N*_E_ | 5.049 | 1.375 | 1.667 | 3.267 | 1.241 | 4.878 | 5.014 | 3.327 | 2.188 |
|  | *H*_O_ | 0.842 | 0.211 | 0.421 | 0.263 | 0.105 | 0.737 | 0.316 | 0.421 | 0.316 |
|  | *H*_E_ | 0.802 | 0.273 | 0.400 | 0.694 | 0.194 | 0.795 | 0.801 | 0.699 | 0.543 |
|  | *HW* | 0.598 | 0.591 | 0.945 | **0.000** | **0.000** | 0.500 | **0.000** | 0.382 | **0.000** |
|  | *F*_IS_ | -0.050 | 0.228 | -0.052 | 0.621 | 0.457 | 0.073 | 0.606 | 0.398 | 0.418 |
| 6 (Mochdre) | *N*_A_ | 12 | 4 | 5 | 3 | 3 | 12 | 7 | 10 | 5 |
|  | *N*_E_ | 7.293 | 1.473 | 1.774 | 2.533 | 1.667 | 6.685 | 5.870 | 5.641 | 4.034 |
|  | *H*_O_ | 0.947 | 0.158 | 0.368 | 0.579 | 0.105 | 0.895 | 0.263 | 0.211 | 0.316 |
|  | *H*_E_ | 0.863 | 0.321 | 0.436 | 0.605 | 0.400 | 0.850 | 0.830 | 0.823 | 0.752 |
|  | *HW* | 0.986 | 0.017 | 0.322 | 0.157 | 0.003 | 0.951 | **0.000** | **0.000** | **0.000** |
|  | *F*_IS_ | -0.098 | 0.509 | 0.156 | 0.043 | 0.737 | -0.052 | 0.683 | 0.744 | 0.580 |
| 7 (Gavenny) | *N*_A_ | 11 | 5 | 4 | 6 | 4 | 11 | 15 | 18 | 5 |
|  | *N*_E_ | 7.577 | 1.756 | 1.436 | 2.735 | 2.683 | 8.087 | 8.715 | 14.017 | 2.808 |
|  | *H*_O_ | 0.724 | 0.345 | 0.207 | 0.621 | 0.241 | 0.793 | 0.379 | 0.414 | 0.241 |
|  | *H*_E_ | 0.868 | 0.430 | 0.304 | 0.634 | 0.627 | 0.876 | 0.885 | 0.929 | 0.644 |
|  | *HW* | 0.004 | 0.325 | 0.015 | 0.868 | **0.000** | 0.132 | **0.000** | **0.000** | **0.000** |
|  | *F*_IS_ | 0.166 | 0.199 | 0.319 | 0.022 | 0.615 | 0.095 | 0.572 | 0.554 | 0.625 |
| 8 (Oregon) | *N*_A_ | 13 | 3 | 3 | 5 | 2 | 13 | 12 | 11 | 3 |
|  | *N*_E_ | 7.848 | 1.174 | 1.112 | 2.473 | 1.670 | 7.848 | 6.333 | 6.685 | 2.571 |
|  | *H*_O_ | 0.842 | 0.053 | 0.105 | 0.474 | 0.000 | 0.842 | 0.684 | 0.368 | 0.000 |
|  | *H*_E_ | 0.873 | 0.148 | 0.101 | 0.596 | 0.401 | 0.873 | 0.842 | 0.850 | 0.611 |
|  | *HW* | 0.673 | **0.000** | 0.996 | 0.017 | **0.000** | 0.673 | 0.005 | **0.000** | 0.007 |
|  | *F*_IS_ | 0.035 | 0.645 | -0.041 | 0.205 | 1.000 | 0.035 | 0.188 | 0.567 | 1.000 |
| 9 (Pant-y-Llyn) | *N*_A_ | 11 | 3 | 4 | 3 | 3 | 8 | 5 | 9 | 3 |
|  | *N*_E_ | 7.237 | 1.336 | 1.296 | 2.288 | 1.300 | 6.169 | 4.034 | 4.404 | 2.972 |
|  | *H*_O_ | 0.813 | 0.219 | 0.125 | 0.375 | 0.000 | 0.719 | 0.310 | 0.188 | 0.406 |
|  | *H*_E_ | 0.862 | 0.251 | 0.229 | 0.563 | 0.231 | 0.838 | 0.752 | 0.773 | 0.664 |
|  | *HW* | 0.249 | 0.236 | **0.000** | 0.056 | **0.000** | 0.335 | **0.000** | **0.000** | 0.003 |
|  | *F*_IS_ | 0.057 | 0.130 | 0.453 | 0.334 | 1.000 | 0.142 | 0.587 | 0.757 | 0.388 |
|  |  |  |  |  |  |  |  |  |  |  |
| MEAN | *N*_A_ | 10 | 3 | 3 | 4 | 3 | 9 | 9 | 10 | 4 |
|  | *N*_E_ | 6.231 | 1.475 | 1.415 | 2.567 | 1.685 | 5.767 | 5.543 | 5.814 | 2.860 |
|  | *H*_O_ | 0.804 | 0.211 | 0.209 | 0.516 | 0.100 | 0.733 | 0.328 | 0.318 | 0.237 |
|  | *H*_E_ | 0.820 | 0.267 | 0.253 | 0.596 | 0.360 | 0.787 | 0.795 | 0.771 | 0.636 |
|  | *F*_IS_ | 0.021 | 0.283 | 0.157 | 0.127 | 0.704 | 0.075 | 0.593 | 0.586 | 0.627 |
|  | *N* | 27.2 | 27.2 | 27.2 | 27.2 | 26.9 | 27.2 | 26.3 | 26.9 | 25.8 |

**Table S4.** Results of ABC analyses for estimated posterior parameters. Means, medians, modes and quantiles are displayed. N1 = Effective population size (*N*_e_) of Sirhowy; N2 = *N*_e_ of Lugg; N3 = *N*_e_ of Dderw; N4 = *N*_e_ of pooled populations (Lea, Mochdre and Gavenny); N5 = *N*_e_ of Bachowey; N6 = *N*_e_ of Pant-y-Llyn; t = time of divergence in generations; Âµmic_1 = mean mutation rate; pmic_1 = number of repeat motifs added/removed from microsatellites in each mutation step and snimic_1 = single insertion nucleotide rate.

| Parameter | mean | median | mode | q025 | q050 | q250 | q750 | q950 | q975 |
| --- | --- | --- | --- | --- | --- | --- | --- | --- | --- |
| N1 | 251 | 219 | 189 | 103 | 116 | 168 | 290 | 463 | 596 |
| N2 | 486 | 382 | 281 | 130 | 156 | 264 | 576 | 1220 | 1610 |
| N3 | 1370 | 1330 | 1240 | 529 | 619 | 1000 | 1710 | 2250 | 2370 |
| N4 | 1270 | 1220 | 1010 | 476 | 564 | 909 | 1590 | 2190 | 2330 |
| N5 | 2460 | 2470 | 2500 | 2370 | 2400 | 2450 | 2490 | 2500 | 2500 |
| N6 | 1050 | 963 | 757 | 327 | 396 | 672 | 1340 | 2060 | 2250 |
| t | 517 | 471 | 417 | 220 | 249 | 368 | 602 | 940 | 111 |
| Âµmic_1 | 0.000925 | 0.000946 | 0.001 | 0.000734 | 0.000774 | 0.000882 | 0.000991 | 0.001 | 0.001 |
| pmic_1 | 0.270 | 0.283 | 0.300 | 0.166 | 0.188 | 0.255 | 0.300 | 0.300 | 0.300 |
| snimic_1 | 0.00000782 | 0.00000958 | 0.00001 | 0.000000583 | 0.00000117 | 0.00000627 | 0.00001 | 0.00001 | 0.00001 |

**Table S5.** Posterior probabilities for all three scenarios estimated with a maximum of 1% of simulated data sets. n = number of simulated datasets closest to the observed using the logistic approach.

| Scenario | n | | | | | | | | | |
| --- | --- | --- | --- | --- | --- | --- | --- | --- | --- | --- |
|  | 3000 | 6000 | 9000 | 12000 | 15000 | 18000 | 21000 | 24000 | 27000 | 30000 |
| 1 | 0.7405 | 0.7903 | 0.8127 | 0.8176 | 0.8179 | 0.8160 | 0.8160 | 0.8145 | 0.8116 | 0.8091 |
|  | (0.4876-0.9933) | (0.6409-0.9396) | (0.7026-0.9229) | (0.7250-0.9102) | (0.7358-0.9001) | (0.7408-0.8913) | (0.7465-0.08854) | (0.7494-0.8797) | (0.7496-0.8736) | (0.7499-0.8684) |
| 2 | 0.0711 | 0.0633 | 0.0650 | 0.0700 | 0.0746 | 0.0795 | 0.0817 | 0.0831 | 0.0844 | 0.0853 |
|  | (0.0000-0.1597) | (0.0077-0.1189) | (0.0176-0.1123) | (0.0256-0.1144) | (0.0323-0.1169) | (0.0385-0.1206) | (0.0428-0.1206) | (0.0462-0.1199) | (0.0493-0.1195) | (0.0518-0.1188) |
| 3 | 0.1884 | 0.1464 | 0.1223 | 0.1124 | 0.1075 | 0.1044 | 0.1024 | 0.1024 | 0.1040 | 0.1055 |
|  | (0.0000-0.4095) | (0.0221-0.2707) | (0.0363-0.2083) | (0.0438-0.1810) | (0.0490-0.1660) | (0.0528-0.1561) | (0.0556-0.1491) | (0.0588-0.1460) | (0.0625-0.1456) | (0.0658-0.1453) |

**Table S6.** StructureSelector output table using the Puechmaille Method to infer the true K (4) from the British *Pacifastacus leniusculus* populations analysed using four alternative statistics (MedMed, MedMean, MaxMed and MaxMean).

| K | MedMed | MedMean | MaxMed | MaxMean | Reps |
| --- | --- | --- | --- | --- | --- |
| 1 | 1 | 1 | 1 | 1 | 20 |
| 2 | 2 | 2 | 2 | 2 | 20 |
| 3 | 3 | 3 | 3 | 3 | 20 |
| 4 | 4 | 4 | 4 | 4 | 20 |
| 5 | 4 | 4 | 4 | 4 | 20 |
| 6 | 4 | 4 | 4 | 4 | 20 |
| 7 | 4 | 3 | 4 | 3 | 20 |
| 8 | 3 | 3 | 4 | 3 | 21 |
|  | MedMedK | MedMeaK | MaxMedK | MaxMeaK |  |
| ALL | 4 | 4 | 4 | 4 |  |

**Table S7.** StructureSelector output table using the Puechmaille Method to infer the true K (5) from the all *Pacifastacus leniusculus* populations analysed using four alternative statistics (MedMed, MedMean, MaxMed and MaxMean).

| K | MedMed | MedMean | MaxMed | MaxMean | Reps |
| --- | --- | --- | --- | --- | --- |
| 2 | 2 | 2 | 2 | 2 | 20 |
| 3 | 3 | 3 | 3 | 3 | 20 |
| 4 | 4 | 4 | 4 | 4 | 20 |
| 5 | 5 | 5 | 5 | 5 | 20 |
| 6 | 4 | 4 | 5 | 5 | 20 |
| 7 | 4 | 4 | 4 | 4 | 20 |
| 8 | 4 | 4 | 4 | 4 | 20 |
| 9 | 4 | 4 | 4 | 4 | 21 |
|  | MedMedK | MedMeaK | MaxMedK | MaxMeaK |  |
| ALL | 5 | 5 | 5 | 5 |  |

**Figure S1**. STRUCTURE analysis for *Pacifastacus leniusculus* populations with ‘null’ microsatellites (Scop1, Scop9, Scop31 and LPL45) removed at K = 5 clusters; each bar represents an individual crayfish, the different colours represent different clusters and therefore indicate the proportion of each crayfish attributable to each cluster. Infection status is stated above the output and corresponding population names stated below for each population.


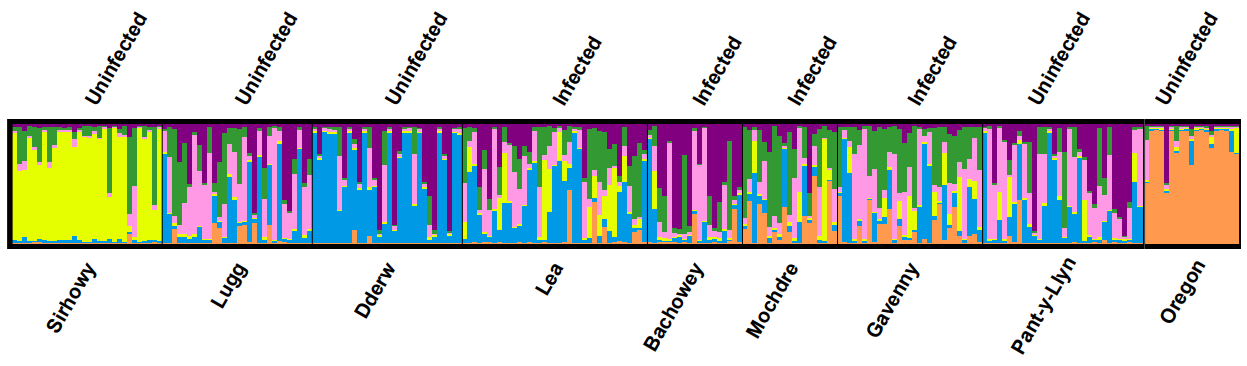

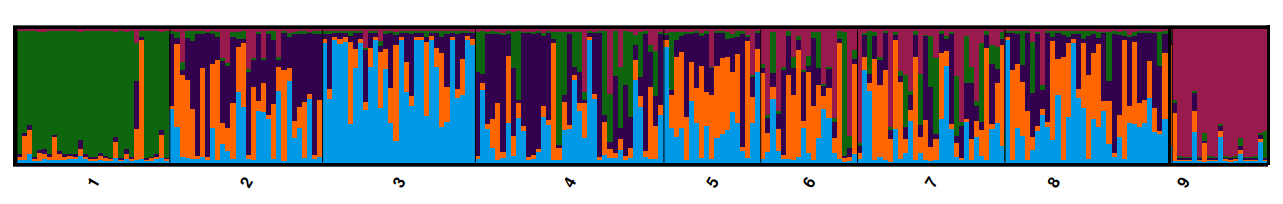

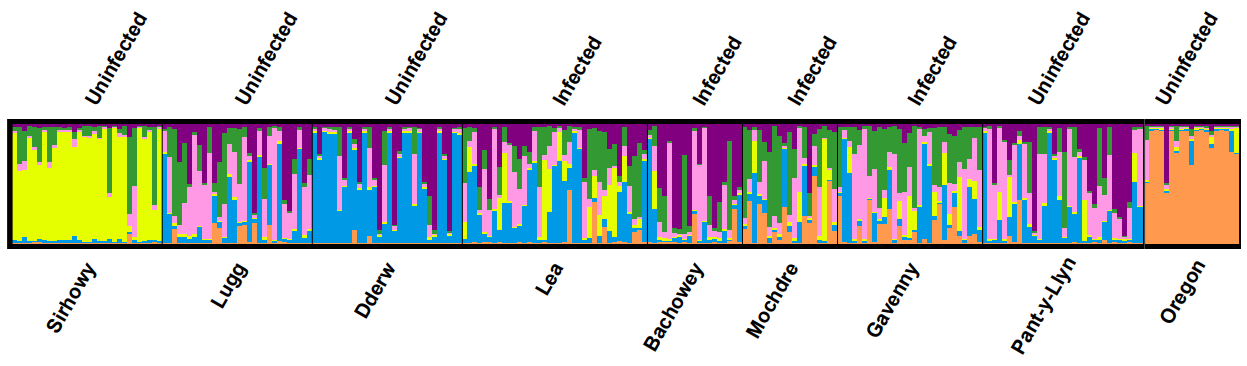


**Figure S2**. Most likely K for all British populations studied (excluding Oregon), determined using MedMedK, MedMeanK, MaxMedK and MaxMeanK statistics in StructureSelector.


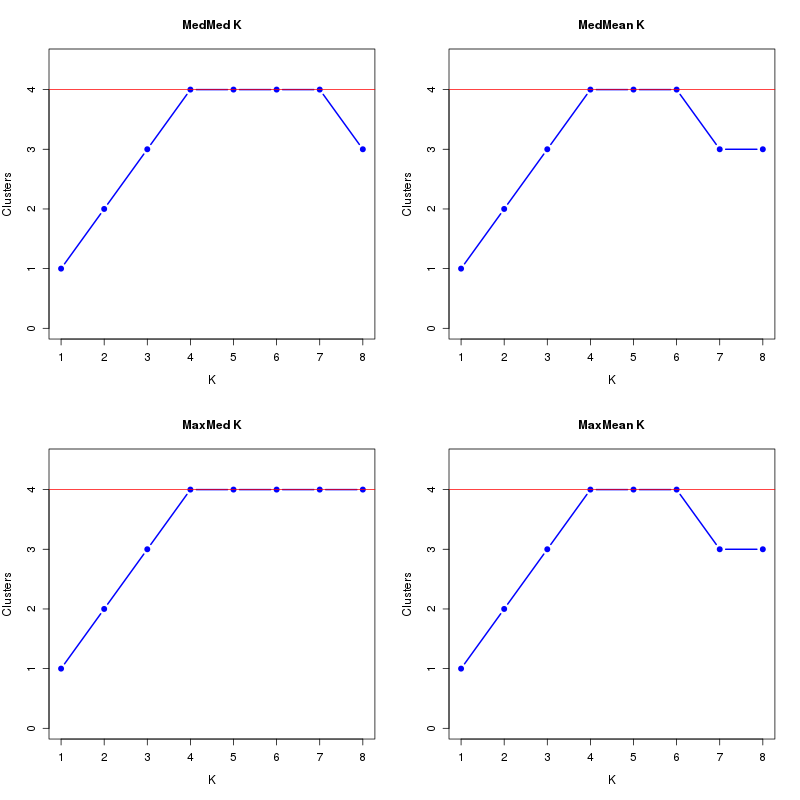


**Figure S3**. Most likely K for all nine populations studied (including Oregon) determined using MedMedK, MedMeanK, MaxMedK and MaxMeanK statistics in StructureSelector.


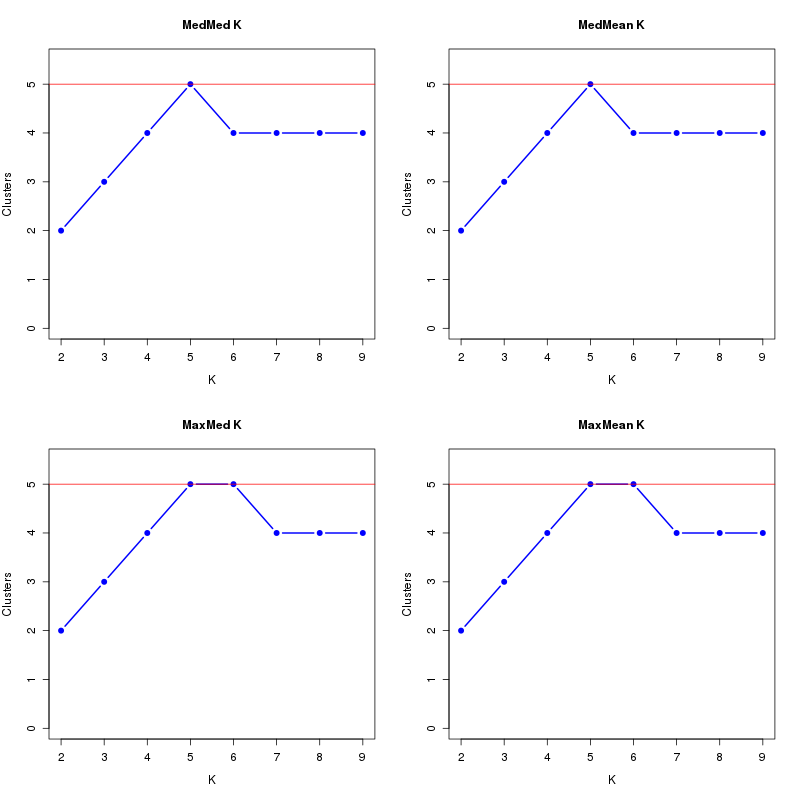


**Figure S4**. Output from BARRIER, highlighting the main breaks in genetic continuity (red lines); the green line corresponds to the Delaunay triangulation between populations (red dots) and Voronoï tessellation used to calculate position of the barriers (blue line). Populations: 1=Sirhowy, 2=Lugg, 3=Dderw, 4=Lea, 5=Bachowey, 6=Mochdre, 7=Gavenny, 8=Pant-y-Llyn. Barriers: a = isolates population 3, b = isolates population 1, c = between population 2 and 5.


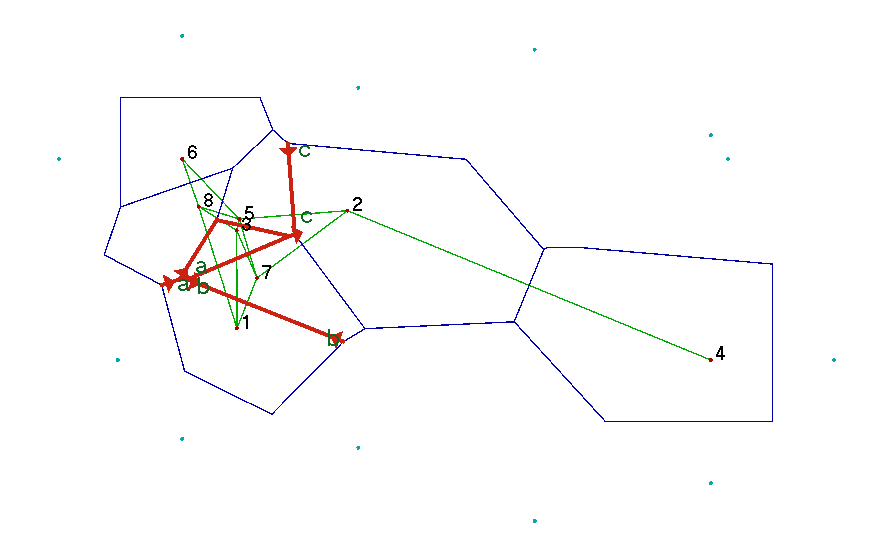


**Figure S5.** Principal component analysis (PCA) showing the fit of posterior distributions for all three scenarios tested, displaying 10000 prior plots per scenario.


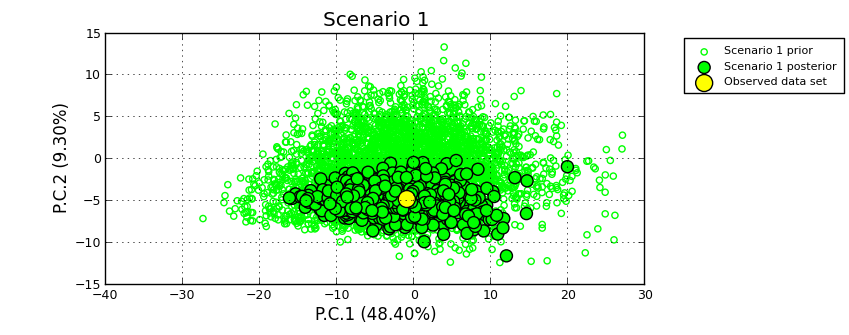

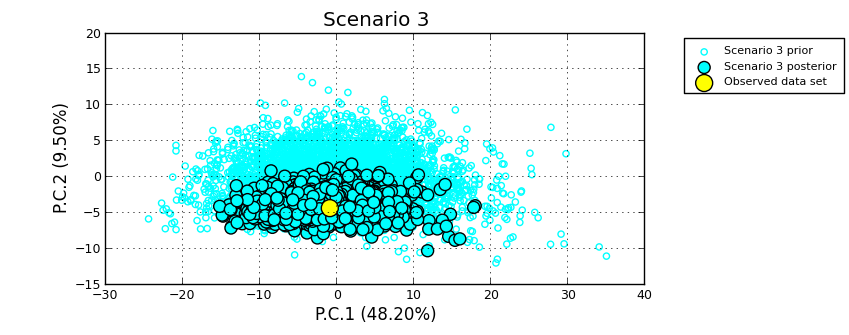

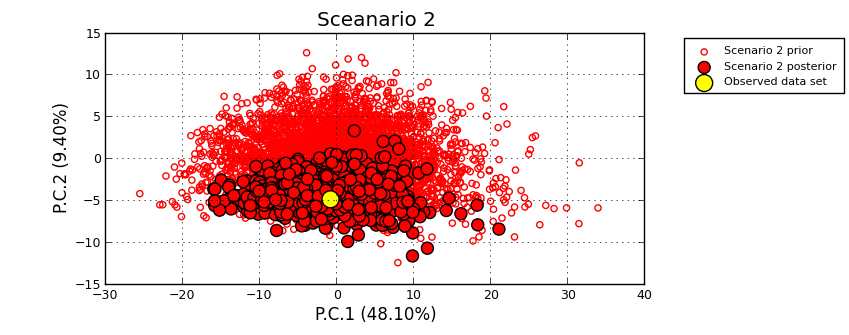

Supplement: Supplementary file 1 [file ECE3-8-9181-s001.docx]
